# Two-Dimensional Layered Double Hydroxides for Reactions of Methanation and Methane Reforming in C1 Chemistry

**DOI:** 10.3390/ma11020221

**Published:** 2018-01-31

**Authors:** Panpan Li, Feng Yu, Naveed Altaf, Mingyuan Zhu, Jiangbing Li, Bin Dai, Qiang Wang

**Affiliations:** 1 Key Laboratory for Green Processing of Chemical Engineering of Xinjiang Bingtuan, School of Chemistry and Chemical Engineering, Shihezi University, Shihezi 832003, China; ppl_19910109@163.com (P.L.); zhuminyuan@shzu.edu.cn (M.Z.); ljbin@shzu.edu.cn (J.L.); db_tea@shzu.edu.cn (B.D.); 2 Environmental Functional Nanomaterials (EFN) Laboratory, College of Environmental Science and Engineering, Beijing Forestry University, Beijing 100083, China; naveed007@bjfu.edu.cn

**Keywords:** layered double hydroxides, two-dimensional materials, methanation reaction, methane reforming, C1 chemistry

## Abstract

CH_4_ as the paramount ingredient of natural gas plays an eminent role in C1 chemistry. CH_4_ catalytically converted to syngas is a significant route to transmute methane into high value-added chemicals. Moreover, the CO/CO_2_ methanation reaction is one of the potent technologies for CO_2_ valorization and the coal-derived natural gas production process. Due to the high thermal stability and high extent of dispersion of metallic particles, two-dimensional mixed metal oxides through calcined layered double hydroxides (LDHs) precursors are considered as the suitable supports or catalysts for both the reaction of methanation and methane reforming. The LDHs displayed compositional flexibility, small crystal sizes, high surface area and excellent basic properties. In this paper, we review previous works of LDHs applied in the reaction of both methanation and methane reforming, focus on the LDH-derived catalysts, which exhibit better catalytic performance and thermal stability than conventional catalysts prepared by impregnation method and also discuss the anti-coke ability and anti-sintering ability of LDH-derived catalysts. We believe that LDH-derived catalysts are promising materials in the heterogeneous catalytic field and provide new insight for the design of advance LDH-derived catalysts worthy of future research.

## 1. Introduction

CH_4_ as a valuable ingredient of natural gas, biogas and coal mine gas plays a prestigious role in C1 chemistry. CH_4_ utilization is an important method to utilize greenhouse gas to protect resources and the environment, and methane catalytically-converted into syngas is a preeminent route to synthesize high added-value chemicals from methane [1,2]. The routes for converting methane to syngas include dry reforming of methane (DRM), steam reforming of methane (SRM), partial oxidation of methane (POM) and autothermal reforming (ATM) [3,4,5,6,7,8]. Among the four kinds of routes for converting methane to syngas, SMR is the most common and renowned economic way to utilize CH_4_ and produce H_2_ and have been applied at the industry scale [9]. Besides, POM can obtain a suitable CO/H_2_ ratio for methanol synthesis and possesses the advantages of high energy efficiency and mild exothermicity, which exhibit great potential in small reactors that are ideal for decentralized applications [10,11,12]. When combining SMR and POM, namely autothermal reforming (ATR), exothermic methane oxidation can provide energy to endothermic steam reforming, thus a large external supply of heat can be avoided. ATR possesses the superiority of easy reactor temperature control and avoids catalyst sintering and carbon deposition, and a wider range of H_2_/CO ratio can be obtained by manipulating the relative concentrations of H_2_O and O_2_, as well [13,14,15]. Furthermore, CH_4_ can also be obtained by CO/CO_2_ methanation, which is a tremendously effectual technology for CO_2_ valorization and processes for coal-derived natural gas production. CO methanation is an effective way to produce CH_4_, and the clean utilization of coal in regions abundant in coal and lacking natural gas has been realized [16,17]. Additionally, CO_2_ methanation plays an important role in the “power to gas” process and also is an efficient method to produce CH_4_ and mitigate CO_2_ emissions, which comprise one of the main sources of global warming [18,19,20,21,22,23]. Layered double hydroxides (LDHs) have a general molecular formula of [M(II)(_1x_)M(III)_x_(OH)_2_]^x+^[A_x/n_^n−^]·mH_2_O, where M(II) represents divalent cations (e.g., Mg, Ni, etc.), M(III) is trivalent cations (e.g., Al, Fe, etc.) and A^n−^ denotes anions [24]. LDHs possess compositional flexibility due to the changeable composition and possess a “memory effect” [25,26,27,28,29,30]. The “memory effect”, i.e., after mild thermal treatment, of LDHs can be reconstructed when contacting the solutions containing various anions [31]. LDH-derived catalysts can form homogeneous mixtures of oxides with a small crystal size, which are stable to thermal treatments and eventually exhibit high thermal stability during high temperature reactions [32]. Due to the high thermal stability and high extent of dispersion of metallic particles, redox stability and Lewis acidity, LDH precursor-derived mixed metal oxides are considered also as qualified supports or catalysts for heterogeneous catalysis.

The active components of catalysts for methanation and methane reforming are similar or equivalent, and the similar reactions are highly exothermic, so conventional supported catalysts easily sinter at high temperatures, leading to catalytic deactivation [33]. The active component can be supported on the surface of LDHs or act as a component of LDHs and form a periclase-like structure to restrain the active component Ni’s agglomeration [34]. LDH-derived catalysts exhibit good thermo-stability and have been widely studied in CO/CO_2_ methanation and methane reforming, which exhibit excellent catalytic performance, as well as anti-coke and anti-sintering abilities. In this paper, a critical review of LDH-derived catalysts for both methanation and methane reforming has been carried out. The catalytic performance, thermal stability, anti-coke and anti-sintering abilities of such catalysts will be discussed assiduously. We believe that the LDH-derived catalysts are promising for the methanation and methane reforming in C1 chemistry.

## 2. Methanation

### 2.1. CO Methanation

The methanation reaction is an ideal way for the coal-derived natural gas production process, for which the catalyst is the key. The reaction of CO methanation is described in Equation (1) [35].

3H_2_ + CO → CH_4_ + H_2_O ΔH = −206.28 kJ mol^−1^ (1)

**Figure 1:**
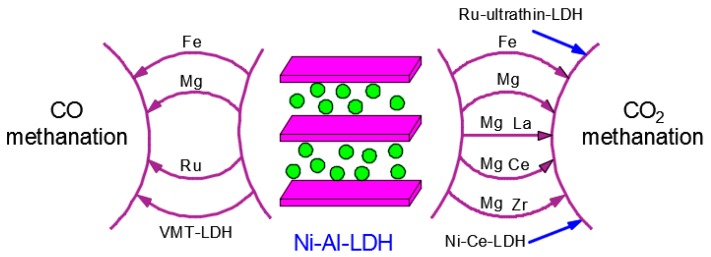
The applications of layered double hydroxides (LDHs) in CO and CO_2_ methanation.

Ni-based catalysts are the most suitable catalysts when taking catalytic performance and cost into consideration [36]. Metallic oxides (Al_2_O_3_, MgO, TiO_2_, ZrO_2_, CeO_2_, etc.) and molecular sieves (MCM-41, SBA-15, etc.) have been employed to act as support for CO methanation catalysts and exhibit good catalytic performance [37,38,39]. However, many traditional supported Ni-based catalysts always possess poor dispersion of the active component and deactivation at high temperatures due to coke formation and active component sintering [40,41]. LDH-derived catalysts show high surface area, uniform metal dispersion and a good thermal property and have been used as catalysts for oxidation of methane, hydrogen production from ethanol and CO methanation, as summarized in Figure 1 [42].

Ni-Al LDH-derived catalysts have been deeply investigated and are well known for CO methanation. In 1994, Rathouskf et al. [43] formulated NiAl-CO_3_ LDH-derived mixed oxide (NiAl-LDO) as the CO methanation catalyst, which maintained excellent activity for the methanation reaction under 2 MPa and 527 °C. The NiAl-LDO catalyst with 56.5 wt % Ni achieved 97% of CO conversion in the pilot methanation unit. Except for the co-precipitation method, the urea hydrolysis method is also an effective route for preparing Ni/Al LDH. Meanwhile, Bian et al. [35] synthesized Ni/Al LDH through the urea hydrolysis method, and the resulting NiAl-LDO catalyst displayed higher catalytic stability due to higher Ni dispersion and stronger resistance to coke deposition compared with the impregnated catalyst. Nearly 100% CO conversion was achieved under reaction temperatures between 400 and 500 °C with a gaseous hourly space velocity (GHSV) of 300,000 mL g^−1^ h^−1^.

However, in the methanation reaction, Ni/Al LDHs also have some drawbacks; for instance, the high nickel content could lead to nickel sintering and carbon deposition during long-term operation [44,45]. Promoters can be released as a drawback, and Hwang et al. [46] reported that the performance of mesoporous nickel-M-alumina xerogel catalysts can be enhanced by introducing a promoter. They found that the yield for CH_4_ decreased in the order of 30Ni10FeAX > 30Ni10NiAX > 30Ni10CoAX > 30Ni10CeAX > 30Ni10LaAX. The 30Ni10FeAX catalyst exhibited the optimal CO dissociation energy and largest H_2_ adsorption ability, which played a key role in determining the catalytic performance, and thus, Fe was regarded as the most suitable second metal component. The 30Ni10FeAX catalyst achieved 99.4% CO conversion and 79.1% CH_4_ yield at 230 °C. Kustov et al. [47] evaluated the influence of the Ni/Fe ratio and the total metal loading on catalytic performance. Two series of mono- and bi-metallic Ni-Fe catalysts were prepared and the catalytic properties tested, among which 25Fe75Ni catalysts were the most active in CO hydrogenation for the MgAl_2_O_4_ support at low metal loadings. The maximum performance of 25Fe75Ni catalysis could be obtained at 20 wt % total metal loading, exhibiting 100% CO conversion and 99.1% CH_4_ selectivity at 275 °C under a GHSV = 50,000 h^−1^. 

**Figure 2:**
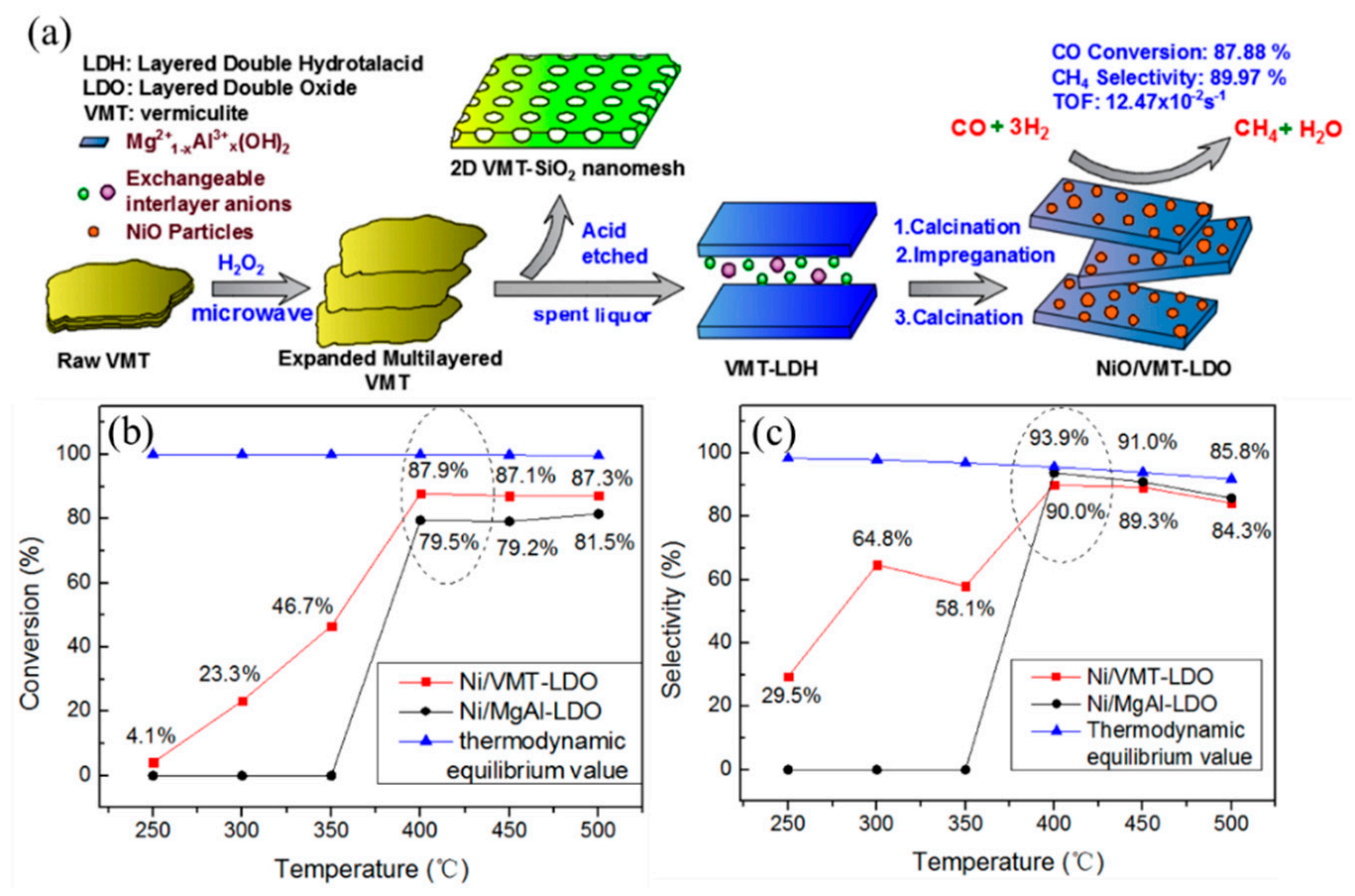
(**a**) Schematic of the preparation process of NiO/VMT-LDO and (**b**,**c**) the catalytic performance of Ni/VMT-LDO [34].

Besides, Mg adulteration can improve the anti-coke ability of Ni/Al LDHs. Li et al. [48] synthesized Ni/Mg/Al LDHs through the co-precipitation method, and the as-obtained NiMg8 (Ni/Mg = 1/8) catalyst with 11 wt % Ni content achieved the best CO methanation performance due to the small size of Ni particles, a higher extent of Ni dispersion and the strong interaction between Ni and MgO and/or Al_2_O_3_ leads to form Ni_x_Mg_1−x_O solid solution during calcination treatment of the Ni/Mg/Al LDH precursor; both properties benefited NiMg8 catalyst, exhibiting an excellent performance. NiMg8 catalyst achieved 99.8% CO conversion and 73.6% CH_4_ selectivity at 550 °C. In our previous work, we have the spent liquor after mixed-acid etching of vermiculite (VMT) (Figure 2), which mainly contained Mg^2+^ and Al^3+^, to synthesize a VMT-derived LDH (VMT-LDH) and prepared Ni/VMT-LDO through the impregnation method. Due to Fe and Ca modification and the improved dispersion of nickel, Ni/VMT-LDO catalyst had smaller Ni nanoparticles than Ni/MgAl-LDO catalyst, leading to better performance than Ni/MgAl-LDO. Compared with Ni/MgAl-LDO, Ni/VMT-LDO catalyst shoed good low temperature activity and achieved 87.9% CO conversion, as well as 90% CH_4_ selectivity at 400 °C [34].

In addition, noble metal doping can not only enhance the NiO reducibility to generate active sites, but also can act as an active component itself, which was favorable for CO methanation. Concurrently, Mohaideen et al. [42] added 1 wt % Ru to NiAl-mixed metal oxides, and the as-obtained catalyst has small Ru particles, which modified the interaction between Ru and Ni, increased the reducibility of NiO and generated more active sites for the CO methanation reaction. Subsequently, Ru/NiAl-C catalysts achieved a CO conversion of almost 100% in the temperature range from 150–220 °C. Catalytic performance of CO methanation for different catalysts in different works were summarized in Table 1.

### 2.2. CO_2_ Methanation

CO_2_ methanation is one of the most eloquent technologies for CO_2_ valorization and played an important role in the “power to gas” process [48]. The equation of CO methanation is as follows [35,49]:

4H_2_ + CO_2_ → CH_4_ + 2H_2_O ΔH = −164.94 kJ mol^−1^ (2)

The active components and supports of catalysts for CO methanation and CO_2_ methanation are similar or equivalent; similar to CO methanation, metallic oxides (Al_2_O_3_, MgO, TiO_2_, ZrO_2_, CeO_2_, etc.) are used as catalysts [21,50,51,52,53,54]. However, similar to the CO methanation catalyst, the main concern is catalyst sintering and carbon deposition, and LDH-derived Ni catalysts present good resistance to coking and sintering under methanation of CO at high temperatures; LDH-derived catalysts are also potential catalysts for CO_2_ methanation.

Similar to CO methanation, Ni-Al LDH was also a commonly-used catalyst. Abate et al. [50] synthesized Ni-Al LDH through the co-precipitation method for CO_2_ methanation. The as-obtained catalyst exhibited better performance compared with commercial catalysts due to the higher metal surface area and metal dispersion. Ni-Al12 catalyst, which was prepared at a pH of 12, has achieved 86% CO_2_ conversion at 300 °C, with a GHSV of 5000 h^−1^. Gabrovska et al. [51] revealed that the sample with Ni/Al = 3 exhibited the highest conversion during all the reactions after reduction at 400 and 450 °C, while the catalyst of Ni/Al = 0.5 surpassed the catalytic performance of Ni/Al = 3 after reduction within 530–600 °C due to the increase of Ni^0^ dispersion.

The precipitation rate and agglomerates varied with hydrophilic colloids, which resulted in diverse metal particle size and further influenced the catalytic performance. The precipitation rate of NaOH, NH_4_OH, Na_2_CO_3_ and (NH_4_)_2_CO_3_ was different; precipitation rate of hydrophilic colloids in Na^+^-based liquors was faster than that in NH^4+^-based liquors; and the metal particle size of the as-obtained catalysts decreased in the order of NiFeAl-NaOH > NiFeAl-NH_4_OH > NiFeAl-Na_2_CO_3_ > NiFeAl-(NH_4_)_2_CO_3_. The increasing order of different catalysts performance is NiFeAl-NaOH < NiFeAl-NH_4_OH < NiFeAl-Na_2_CO_3_ < NiFeAl-(NH_4_)_2_CO_3_ [55]. (Ni,Mg,Al)-LDH-derived catalyst was first used as a CO_2_ methanation catalyst by Bette et al. [56]. The as-prepared catalyst reached a maximum of (74 ± 2)% between 330 and 350 °C. Mg-Al oxide-supported Ni catalyst also displayed better performance than Ni/MgO and Ni/Al_2_O_3_ catalysts in CO + CO_2_ methanation. The stronger interaction between the support and active component led to excellent thermal stability during the CO + CO_2_ methane reaction. Such a catalyst achieved a 98.4% CH_4_ yield at 250 °C and maintained a 95.2% CH_4_ yield at 700 °C for 8 h [57].

In pursuit of further improvement of the anti-coke ability of (Ni,Mg,Al)-LDH-derived catalysts, some dopants were introduced. La adulteration of Mg-Al-Ni LDH-derived catalysts can form a periclase-like structure and new medium strength basic sites, promote the CO_2_ adsorption capacity of the catalysts, soften the interaction between Ni-species and the LDH matrix and improve CO_2_ conversion [58,59]. Furthermore, Wierzbicki et al. [59] investigated the effect of the La incorporation method: Ni_21_La_0.4_(IE) catalyst prepared by ion-exchange displayed the best catalytic performance and achieved ~80% CO_2_ conversion at 300 °C, while the impregnation led to a decrease in the amount of medium strength basic sites, while the catalytic performance of Ni_21_La_1.1_(IMP) had no obvious advantage compared with the Ni_21_ catalyst. In Nizio et al.’s [60] survey of LDH-derived materials, Ce or Zr adulteration cannot improve the catalytic activity, even though the addition of Ce or/and Zr improved the total basicity. In the hybrid plasma-catalytic methanation, the HTNi catalyst exhibited its activity around 80% conversion at 110 °C, whereas only at relatively high temperatures, Ce-promoted catalysts can show interestingly high activities. Moreover, the author thought the medium-sized zero-valent Ni crystallites of non-promoted HTNi (15 nm) catalyst seemed to be more active during off plasma methanation than far too small ones (8.1 nm of HTNi-CeZr), whereas the influence of the basicity of catalysts on their activity remains relatively unclear. However, plasma-assisted Ni-Ce-LDH-P synthesized catalyst reported by Xu et al. [61] in 2017 exhibited better performance compared to HTNi-Ce; Ni-Ce-LDH-P achieved almost 75% CO_2_ conversion at 270 °C due to the smaller Ni size, better Ni dispersion and higher alkalinity, whereas Ni-Ce-LDH-C catalyst exhibited the same CO_2_ conversion at 300 °C. Likewise, characterization results revealed that the precursor of Ni-Ce-LDH-P catalyst presented a lamellar shape, implying the formation of chemical bonds among Ni, Ce and Al (from Al_2_O_3_). Actually, it was the chemical bonds that improved the dispersion of Ni crystal and the interaction between Ni and γ-Al_2_O_3_. Meanwhile, the plasma technology with a relatively low temperature prevented the sintering and agglomeration of Ni during the preparation process.

**Figure 3:**
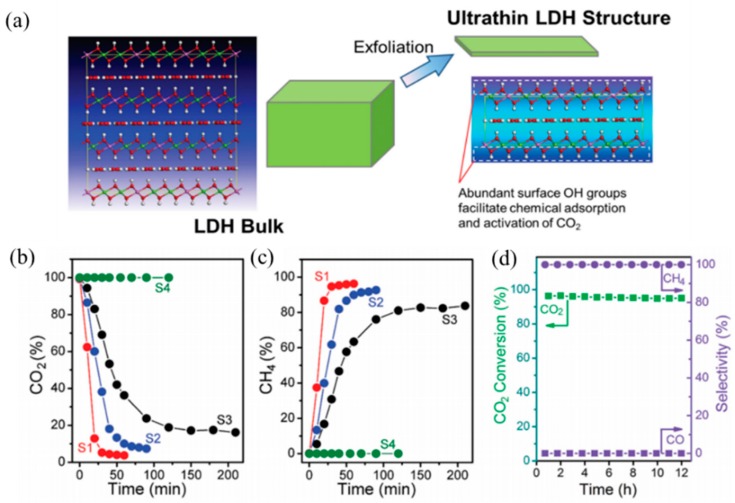
(**a**) Schematic presence of the formation of the ultrathin LDH structure and (**b**–**d**) the catalytic performance of Ru@FL-LDHs [64].

When the thickness of the material was reduced to the atomic monolayer, the electron density increased, which benefited the high-speed transfer of carrier in the material. This property enabled two-dimensional materials to exhibit great potential in heterogeneous catalysis [62]. Single-layer LDHs (SL-LDHs) drew much more attention recently because of their high performance as energy materials, such as exfoliated NiCo, CoAl and NiFe LDHs [63]. Ren et al. [64] prepared a Ru-loaded ultrathin LDH through ultrasonic exfoliated commercial LDHs in 2016, and the AFM (atomic force microscopy) image showed that the thicknesses of these ultrathin structures were around 8 Å, which nearly corresponds to a single basal spacing of the LDH crystals (Figure 3). The as-prepared catalyst had similar wrinkles to graphene, and curls existed at the edge and exhibited almost monodispersed Ru nanoparticle or small Ru-nanoparticle aggregates. Ru@FL-LDHs catalyst had the strongest light absorption and exhibited an excellent catalytic performance. Ru@FL-LDHs achieved the highest CO_2_ conversion of about 96.3% and 99.3% of selectivity toward the CH_4_ in photocatalytic CO_2_ methanation.

In conclusion, LDH-derived Ni catalysts were more favorable than traditional impregnated catalysts, while their study in the methanation reaction was limited. As promising catalysts for the methanation reaction, more attention should be paid to future improvement of the catalyst activation and stability, and the reaction mechanism of this kind of catalyst is also worthy of research. Catalytic performance of CO_2_ methanation for different catalysts in different works were summarized in Table 2.

## 3. Methane Reforming

### 3.1. Dry Reforming of Methane

**Figure 4:**
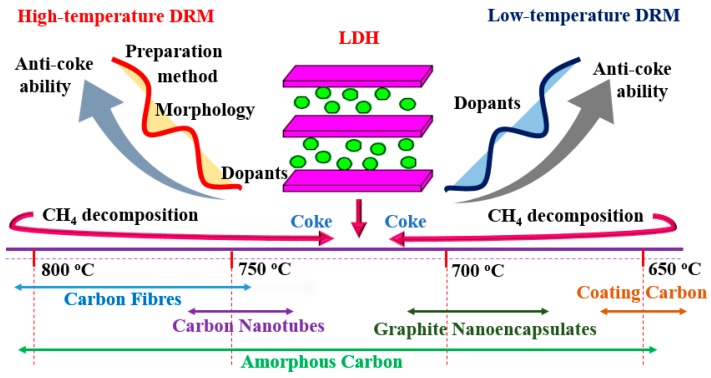
The application of LDHs in dry reforming of methane. DRM, dry reforming of methane.

Dry reforming of methane (DRM) is a critical method for obtaining added-value products from CO_2_ and an effective way to utilize these two greenhouse gases. LDHs are also widely investigated in dry reforming of methane (Figure 4) [6,7]. The equation for the dry reforming of methane (DMR) is as follows [21]:

CH_4_ + CO_2_ → 2CO + 2H_2_ ΔH = 247.3 kJ mol^−1^ (3)

Likewise, two reaction mechanisms of DMR have been surveyed. One mechanism was the Eley–Rideal-type mechanism [65,66]. Methane is firstly adsorbed on the metal and decomposed to H_2_ and adsorbed carbon. Then, the adsorbed carbon is reacted directly with CO_2_ to yield CO. The equation is as follows:

CH_4_ → C(s) + 2H_2_ (4)

C(s) + CO_2_ → 2CO (5)

Additionally, as an alternative reaction mechanism [67,68,69,70], methane was decomposed on the metal and produced surface CH species and hydrogen; carbon dioxide molecules decomposed to CO; and oxygen (O(s)) adsorbed at the same time. Afterwards, adsorbed oxygen and CH species reacted to give CO and H_2_. The equation is as follows:

CH_4_ → CHx(s) + (4 − x)/2H_2_ (6)

CO_2_ → CO + O(s) (7)

CHx(s) + O(s) → (x/2) H_2_ + CO (8)

#### 3.1.1. High Temperature Dry Reforming of Methane

Noble metals like Ru reflected excellent catalytic performance and anti-coke deposition ability in the DRM reaction. Supports have great influence on the dispersion of the active component Ru. Ru metal dispersed on different supports followed the order: Ru/Mg_3_(Al)O > Ru/MgO > Ru/MgAl_2_O_4_ > Ru/γ-Al_2_O_3_. Ru/Mg_3_(Al)O catalyst showed higher catalytic performance due to the strong base intensity of support and more surface Ru^0^ atoms, which ascribed to Mg(Al)O mixed oxide’s unique properties, including memory effect, low crystallinity and its strong interaction with Ru [71]. Ru/Mg_3_(Al)O catalyst displayed an 84% CH_4_ conversion during the 30-h stability test without any deactivation. 

Even though noble metal-based catalysts exhibited excellent catalytic performance, industrial applications were limited due to the high cost. Ni was a suitable active catalyst component when taking catalytic performance and cost into consideration. In 1988, Bhattacharyya et al. [72] investigated the performance of LDH-derived catalysts in CO_2_ reforming of methane. Compared with commercial Ni/Al_2_O_3_ catalysts, the LDH-derived Ni_4_Al_2_O_7_ catalyst showed identical performance at 815 °C and 2.07 MPa. LDH-derived catalysts had superior stability and a coke-resistant ability according to aging studies, and the LDH-derived Mg_5_NiAl_2_O_9_ catalyst exhibited the highest CH_4_ conversion of 95.8% under operation conditions at 850 °C, 0.67 MPa, GHSV = 14,400 h^−1^ and CO_2_/CH = 1.25; while Touahra et al. [73] discovered that Ni/Al = 2 was optimal for Ni-Al LDH-derived catalysts due to the low Ni^0^ crystallite size and high stability of the support (NiAl_2_O_4_).

**Figure 5:**
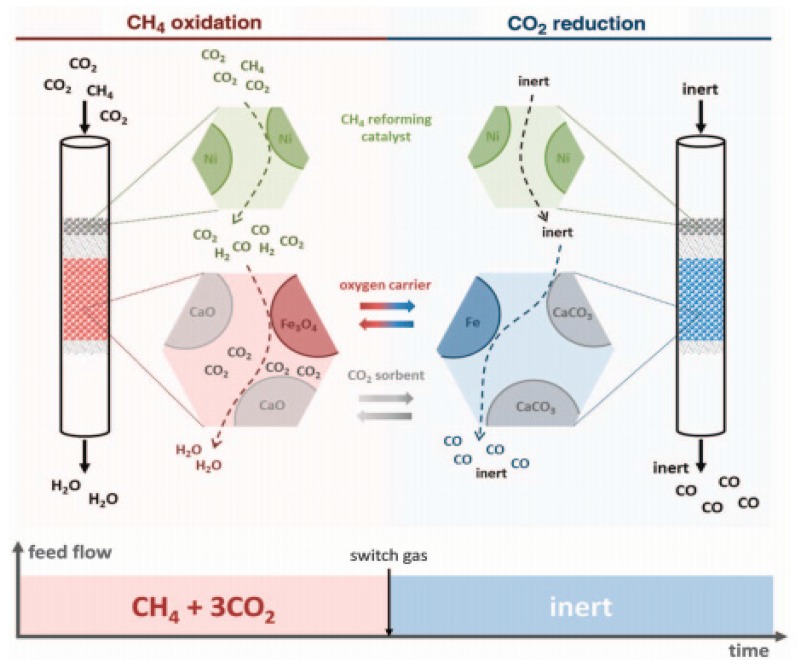
Schematic representation of the “super dry” reforming process [81].

Consequently, when Mg was introduced to Ni-Al LDHs, the catalytic performance and anti-coke ability of the catalyst improved. Mette et al. [74,75,76] found that Ni/MgAlOx catalyst has good anti-coke ability, and the catalytic performance revealed no decrease in performance after 19 times of cycling because nickel aluminate overgrowth on the Ni particles blocked all extended metallic Ni sites, which were nucleation centers for carbon formation. As reported by Lopez et al. [77], the catalytic properties of Ni-Mg-Al catalysts were more affected by the M^II^/M^III^ ratio compared with the Ni/Mg ratio. When M^II^/M^III^ was maintained, the catalyst activity was related to the nickel crystal size and Ni/Mg ratio, while selectivity suffered little from the Ni/Mg ratios. This notwithstanding, when Ni/Mg was constant, the catalyst activity was strongly affected and decreased as the M^II^/M^III^ ratio decreased. Besides, Zhu et al. [78] showed that NiMgAl catalyst with a Mg/Al ratio of 1 displayed the best activity and stability during the DRM reaction, and it was the formation of LDH precursors and MgNiO_2_ that played the key role in stabilization. Interestingly, Li et al. [79] demonstrated that the performance of Ni/Mg(Al)O catalyst decreased at the beginning due to the MgO film surrounding the Ni particles; however, the catalyst was renewed after MgAl_2_O_4_ spinel-like phase formation. TheHT-700 catalyst reached approximately 95% CH_4_ conversion after 500 h of reaction and maintained for 1500 h. In 2016, Buelens et al. [80] developed a “super-dry” CH_4_ reforming through Le Chatelier’s principle reaction (Figure 5): Ni/MgAl_2_O_4_ was used as the catalyst during the “super-dry” CH_4_ reforming. Fe_2_O_3_/MgAl_2_O_4_ was used as the solid oxygen carrier, which oxidized CH_4_ into CO_2_ and H_2_O. Meanwhile, the Fe_2_O_3_ reduced to Fe; CaO/Al_2_O_3_ was used as the CO_2_ sorbent, which formed CaCO_3_ and then decomposed into CaO and CO_2_, and CO_2_ reduced to CO by Fe through a redox reaction. “Super-dry” CH_4_ reforming also resulted in a very low exergy destruction per mole CO_2_ converted; the exergy destruction for CO_2_ conversion was up to 25–50% lower as compared with that of conventional DRM. “Super-dry” CH_4_ reforming can result in higher CO production and showed both practical and economic benefits compared with conventional dry reforming.

**Figure 6:**
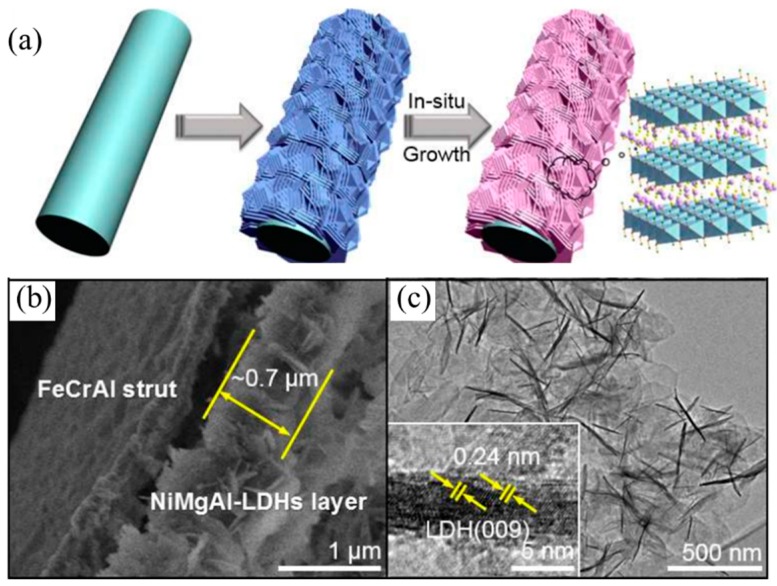
(**a**) Schematic of microfibrous-structured NiO-MgO-Al_2_O_3_ nano-sheets grown on FeCrAl-fiber felt and (**b**) SEM image and (**c**) TEM image of NiMgAl-LDHs/FeCrAl-fiber-900 [82].

Besides the classic co-precipitation method, other methods also have been employed to prepare high efficiency Ni/Mg/Al LDH-derived catalysts. Meanwhile, Shishido et al. [81] investigated the influence of the preparation method on the catalytic performance of Ni/Mg-Al catalyst. Likewise, the solid phase crystallization (spc) method can promote Ni^2+^ replacement of the Mg^2+^ site in LDH, resulting in the formation of highly dispersed 7% Ni metal particles, while the Ni dispersion of imp-Ni/Mg-Al was 4.8% (prepared by impregnation method). The catalyst spc-Ni/Mg-Al exhibited a slightly better catalytic performance than imp-Ni/Mg-Al at 800 °C. In addition, Chai et al. [82] developed a FeCrAl-fiber-structured nanocomposite NiO-MgO-Al_2_O_3_ catalyst in one step, using a γ-Al_2_O_3_/water interface-assisted method, as shown in Figure 6. The as-obtained catalyst exhibited excellent stability due to Ni nanoparticles being uniformly nested in MgO-Al_2_O_3_ nano-sheet composites after reducing of the catalyst. Difficult carbon deposition was the main cause for catalyst deactivation, and the deactivation rate was significantly decreased as compared with the Ni/Al_2_O_3_ catalyst. The NiO-MgO-Al_2_O_3_ catalyst achieved a CH_4_ conversion of 91% at 800 °C with a GHSV of 5000 mL g^−1^ h^−1^, CH_4_/CO_2_ = 1.0/1.1. In situ growth of LDH on γ-Al_2_O_3_ is also an effective way to prepare DMR catalysts. The NiMgAl-LDO/γ-Al_2_O_3_ catalyst has a small Ni nanoparticles size, a strong metal-support interaction and finely-tailored surface basicity. The NiMgAl-LDO/γ-Al_2_O_3_ catalyst achieved an 80.7% CH_4_ conversion at 700 °C and showed outstanding stability during the 48-h test [83]. Zhang et al. [84] reduced LDH by atmospheric cold plasma jet, and the as-obtained C-LDHs/γ-Al_2_O_3_ catalyst can avoid the side-reaction in CO_2_-CH_4_ reforming, leading to better carbon deposition resistance. C-LDHs/γ-Al_2_O_3_ catalyst achieved the same CH_4_ conversion of 98% as Ni/MgO/γ-Al_2_O_3_ catalyst at 800 °C, but had higher H_2_ selectivity.

Besides, the main challenge for NiMgAl LDH-derived catalysts was carbon deposition, and many efforts have been made to overcome this obstacle, such as utilizing metal adulteration and changing the morphology of the catalyst. Noble metal Rh as a promoter for Ni catalysts can improve the catalyst activity; Rh adulteration increased the amount of reducible Ni and promoted the dispersion of Ni. In addition, the presence of Rh probably led to Ni segregation with time on stream and generated carbon deposition due to Rh having favorable CH_4_ decomposition [85]. Moreover, Zr adulteration to LDH can form periclase-like mixed oxide and rearrangement of Ni particles during the DRM reaction. The HNiZr_3_ catalyst exhibited the highest coking resistance properties due to Zr species in the lattice of periclase-like mixed oxide, resulting in small Ni crystallites, which are inactive in direct methane and Boudouard reaction oxides. Yet, Zr adulteration decreased the activity, and the CH_4_ conversion of HT-25Ni catalyst was 40% and 23% for HTNi-Zr catalyst [86]. La as a promoter can improve nickel metal dispersion and increase the total amount of basic sites and surface Ni content [87]. With 1.1 wt % La adulteration, the catalyst properties vary as the Mg/Al molar ratio changes, and the higher Mg/Al molar ratio enhanced the catalyst stability with fewer carbon deposition, but decreased activity. The best stability was achieved at Mg/Al = 3 during a 120-h test, and the hydro-magnesite phase was formed with a Mg/Al molar ratio of four [88,89,90,91].

Ce as a promoter has been revealed by Daza et al. [92,93,94], who explored two adulteration methods. Ce promoted by the co-precipitation method at constant pH formed mixed reducible phases of the NiO-MgO (periclase) type and CeO_2_ (fluorite), and the as-obtained catalyst exhibited better performance than the un-promoted catalyst. OM_2_ (Ce-NiMgAl-LDO) catalyst with 14.9% CeO_2_ displayed about a 75% CH_4_ conversion during the 200-min reaction, and the CO_2_ conversion was also about 10% higher than that of OM1 (NiMgAl-LDO) catalyst, which contained no CeO_2_. Further study revealed that Ce and Mg had a synergic effect on the CO_2_ adsorption capacity of the solids, promoted the basicity of oxides and enhanced their catalytic activity in CO_2_ reforming of methane. However, high loads of Ce decreased the superficial area of the solid and favored the formation of free NiO, which had a negative impact on the selectivity and increased the formation of coke. The fact-finding of Djebarri et al. [95] insinuated that the NiMgCe catalyst was a very stable, and a poorly-reducible mixed oxide phase was formed with Ce adulteration, which inhibited the catalysts’ catalytic performance. Besides, the Ce addition affected the catalytic performance. Ren et al. [96] unveiled that Ce introduced through the incipient impregnation method showed higher Ce^3+^ content and appropriate interactions between Ni and NMA compared with catalyst prepared by the co-precipitation method. Daza et al. [97] also revealed that with Ce introduced by partial reconstruction, the as-prepared catalyst formed periclase and fluorite mixed phases after the calcination, and the reconstruction took place at the external edges of the oxide granules. Ce presented an improvement in the degree of reduction of Ni, the amount and strength of the basic sites. With Ce loading increased, no obvious considerable differences in the catalytic activity and selectivity were perceived, but the anti-coke ability was improved. The optimal amount of Ce doping was 3 wt %, and OM3 (Ce-NiMgAl-LDO) catalysts with (Ni + Mg)/Al = 3) achieved a 90.3% CH_4_ conversion at 700 °C, with the CO_2_/CH_4_/He ratio of 20/20/60 and weight hourly space velocity (WHSV) = 48 L g^−1^ h^−1^.

Additionally, the anti-coke ability can also be improved through incorporating carbon in the Ni-based catalyst. In 2017, Jin et al. [33] used sucrose as the carbon source to incorporate carbon in the Ni-based catalyst, and carbon incorporation formed new mesopores, increased the specific surface area and inhibited Ni particle growth. The as-prepared PC-350-1.8 (pretreated catalyst) catalyst exhibited relatively lower initial conversions of CH_4_ and CO_2_ than pure Ni-LDO catalyst, but showed excellent anti-coke ability. The R-C-350-1.8-800 catalyst was obtained by removing the upper carbon from PC-350-1.8 catalyst, displaying 10% higher CH_4_ conversion and showing slightly lower carbon deposition.

**Figure 7:**
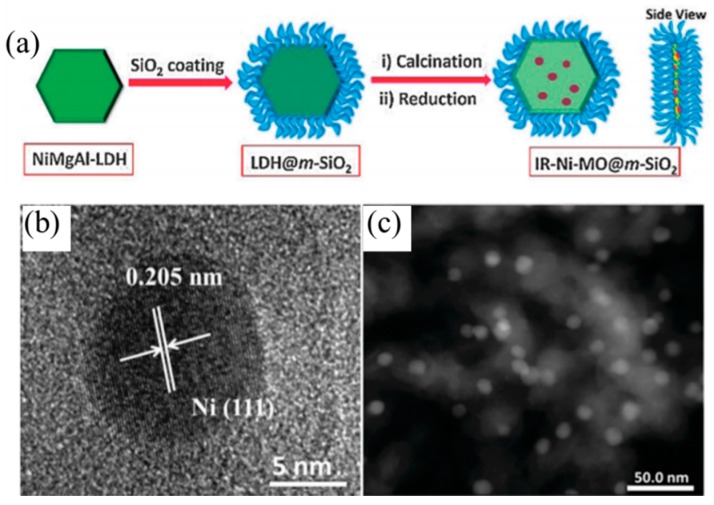
(**a**) Schematic illustration of the preparation process of the modular catalysts. (**b**) TEM image and (**c**) HAADF-STEM image of the modular catalysts [99].

Besides transition elements and rare-earth elements, the advantage of the coke resistance ability changed the morphology of the catalysts and can also promote the anti-carbon deposition ability. Du et al. [98] synthesized monolithic Ni-Mg-Al LDH catalyst nanosheets via in situ growth on Al wires. The as-prepare catalyst showed a hierarchical porous structure, and the oxide nanosheets were arranged as a dense film on the aluminum substrate. Monolithic catalysts showed strong metal-support interactions and strong basic sites compared with traditional Ni-MgO-Al_2_O_3_ catalysts. The monolithic catalysts have also displayed excellent sintering resistance and anti-carbon deposition ability, and coke deposition on the monolithic catalysts was one third that on the traditional catalysts. Du et al. [99] also developed a modular catalyst by combining the Ni-MgO-Al_2_O_3_ mixed oxide nanoplates with the mesoporous SiO_2_ coating, the as-prepared NiMgAl-LDH@m-SiO_2_ catalyst core shell structure (Figure 7) and the dual confinement effects: The first confinement was MgO, which can promote embedded Ni NP dispersion and enhance the chemisorption ability of CO_2_, as well as restrain the carbon deposition and Ni NP aggregation. The second confinement resulted from the mesoporous SiO_2_ shell, which exhibited another “confinement effect”. These two confinement effects can reinforce each other, which enabled the modular catalysts to show excellent anti-coke and sintering resistance ability. IR-NiMgAl-LDH@m-SiO_2_ achieved a 90% CH_4_ conversion at 800 °C, much higher than that of the IM-NiMgAl-LDH@m-SiO_2_ (~55%) catalyst obtained by the impregnation method. Gonzáleza et al. [100] made a Ni-Mg-Al nano-spheroid oxide catalyst through the sol-gel method, and this nano-spheroid oxide catalyst with 15 wt % Ni achieved a 95% CH_4_ conversion at 800 °C and showed excellent long-term stability. Amorphous carbon formed on the nano-spheroid catalyst surface during the reaction, which seemed not to be detrimental to this reaction. Encapsulation of carbon is the main culprit in the nickel-containing catalyst’s deactivation, because the nickel crystallites are encapsulated by the carbon. However, for this nano-spheroid catalyst, the majority of the carbon was amorphous, and a few seeds of encapsulated carbon benefited from the good conformation of the active sites formed by the nano-crystalline structure of the mixed oxides Mg(Al,Ni)O. In this regard, combining Mg-Al mixed oxides with SBA-15 is also an effective way to improve the anti-coke ability. Zuo et al. [101] found that the catalyst whose metal oxides were calcined two times showed excellent anti-carbon deposition and catalytic stability, due to the strong metal-support interaction and the channel local effect of SBA-15. The SH-550 (SBA-15 added to the hydrotalcite suspension during the preparation) and HS-550 (hydrotalcite added to the SBA-15 suspension during the preparation)catalysts, for which the metal oxides have been calcined two times, achieved almost an 85% CH_4_ conversion and maintained excellent stability at 800 °C. For the HS-550 catalyst, the Ni^0^ particles were isolated from one another, so that the clustering of Ni, which is necessary for coke formation, was prevented. In addition, the HS-550 catalyst contained two nickel species: Ni^0^ and NiO; the coexistence of these two species favored the high catalyst activity reported by Damyanova et al. [102].

Furthermore, Co was also an active component of DRM reforming, and Co containing LDH has been used as the DRM catalyst. Liu et al. [103] discovered that the catalytic activity, stability and coke resistance of Co/MgAl increased with the increase of Co loading, and the 12% Co/Mg_3_Al catalyst showed much higher catalytic stability and much less coke deposition at 600 °C as compared to the 12% Ni/Mg_3_Al catalyst, which may be attributed to the higher affinity of Co for oxygen species; in addition, Co has a higher interaction with support. The results suggested that Co has the potential compared to Ni to be an active catalyst in the CH_4_-CO_2_ reforming reaction. In Gennequin et al.’s study [104], the Co_2_Mg_4_Al_2_HT500 catalyst exhibited better stability as compared with the Co_4_Mg_2_Al_2_HT500 catalyst, though the Co_2_Mg_4_Al_2_HT500 catalyst has a lower initial activity. The Co_6_Al_2_HT500 catalyst showed comparable catalytic performance to the Co_2_Mg_4_Al_2_HT500 catalyst during the stability test, while Co_6_Al_2_HT500 showed a larger amount of deposited carbon. After the reaction, three catalysts exhibited a weight decrease corresponding to carbon oxidation following the order: Co_6_Al_2_HT500 (−52%) > Co_4_Mg_2_Al_2_HT500 (−23%) > Co_2_Mg_4_Al_2_HT500 (−5.5%). Mg oxides could inhibit coke deposition via adsorbed CO_2_ species on the basic site and afterwards react with the deposited carbon by the reverse Boudouard reaction. The Co_2_Mg_4_Al_2_HT500 catalyst has a higher Mg content, thus showing excellent anti-coke deposition stability. It could be concluded that for the reaction of the dry reforming of methane, the catalytic performances of CoxMgyAl_2_HT500 solids have a great relation to Co and Mg content. Gennequin et al. [105] used the “memory effect” of LDHs to impregnate the 1 wt % Ru into CoxMgyAl_2_, and the as-obtained catalyst exhibited better performance than the conventional impregnation method in the temperature region of 450–750 °C. Regarding this, small amounts of Ru can promote the reducibility of cobalt catalysts and enhance the stability of the catalysts via decreased carbon formation; the catalyst obtained by the “memory effect” generated both metallic and basic sites, which are favorable for the dry reforming reaction of methane.

Moreover, Ni-Co bimetallic LDHs were prepared by the in situ synthesized method on the surface of γ-Al_2_O_3_. The as-obtained catalysts showed a strong interaction between the active component (Ni and Co) and the catalytic support [106]. However, Tanios et al. [107] discovered that the Ni and Co synergistic effect greatly improved the catalytic properties and prevented carbon formation. The 1Co-2Ni-LDH catalyst exhibited the best catalytic performance and resulted in the least coke, achieving a 98.3% CH_4_ conversion that decreased to 1.7% after 50 h of reaction at 800 °C, much higher than that of Co/γ-Al_2_O_3_, which resulted in a 56.2% CH_4_ conversion and complete deactivation after the 50-h test; while the optimal Co/Ni ratio was one when using aluminum nitrate as the trivalent ion source.

#### 3.1.2. Low Temperature Dry Reforming of Methane 

The DRM reaction was always performed at high temperatures to obtain a better performance, whereas LDHs as the catalyst precursors for low temperature dry reforming of methane were studied by Debek et al. [108]. Ni-Al LDH was firstly used as the catalyst for the dry reforming of methane. The HT-NiAl catalyst reduced at 900 °C exhibited a 48% CH_4_ conversion and about a 55% CO_2_ conversion, higher than that of the HT-NiAl catalyst reduced at 550 °C, due to the reduction temperature of 550 °C not being sufficient to reduce all of the nickel species to being metallic. Moreover, the side reaction of CH_4_ decomposition occurring due to an excess of H_2_ was observed, and a ca. 30% conversion of methane was obtained by CH_4_ on decomposition catalysts reduced at 550 and 900 °C, which evinced that methane decomposition strongly influences the overall process; in addition, the CH_4_ decomposition was not influenced by the temperature of the catalyst’s pre-treatment.

As the active component, the incorporation method and the content of Ni have a great effect on the DRM reaction. Higher values of CH_4_ and CO_2_ conversions were obtained for the sample (HTNi) prepared by the co-precipitation method with 63.47 wt % Ni content. About a 55% CH_4_ conversion was obtained at 550 °C for the HTNi catalyst, and additional catalytic tests were performed on CH_4_ decomposition on the sample HTNi at 550 °C with a feed gas of CH_4_/Ar = 2/8. However, HTexNi catalyst with 0.78 wt % Ni displayed higher activity per gram of active material, due to the formation of small Ni NPs or aggregates of nickel oxide on the catalyst surface [109]. Ni particle size increased with Ni content increasing, and dry reforming of methane and direct methane decomposition showed increased methane conversion. Methane decomposition and carbon formation may occur, especially in the presence of catalysts that contain Ni in considerable amounts, and the catalysts with different Ni contents had the same catalytic performance trend as the DRM reaction. Thus, methane decomposition at low temperatures can be controlled by decreasing the Ni particle/crystal size [110].

Because the side reaction of methane decomposition was inevitable during the DRM reaction, methane decomposition led to carbon deposition and catalyst deactivation. Daza et al. [97] found that Ce-promoted catalysts had excellent coke resistance ability. Debek et al. investigated the route cause for this phenomenon. Bigger Ni crystallites on the catalysts with the highest Ni content promoted direct CH_4_ decomposition and accelerated catalyst deactivation. Ce-promoted Ni-containing LDH suppressed the side reaction of CH_4_ decomposition due to its high basicity enhanced CO_2_ adsorption and excellent ability to oxidize the already formed carbon deposits, whereas too high a loading of ceria had a negative effect during the overall process due to the formation of free NiO [111].

Meanwhile, Zr doping can further promote the coke resistance ability of catalyst and substantially restrain the extensive formation of fishbone-type carbon nanofibers. With the adulteration of Zr, the CH_4_ conversion decreased, but Zr considerably inhibited methane’s direct decomposition, favored methane reaction with CO_2_ (DMR reaction), together with other important parallel reactions, such as the reverse Boudouard reaction. The HT-25Ni catalyst achieved 48% CH_4_ conversion and showed 25% CH_4_ conversion at 550 °C; even though both CH_4_ and CO_2_ conversions of the HTNi-CeZr catalyst were low, almost no carbon was deposited on the catalyst surface during 5 h of DMR reaction [112]. The catalysts with Ce/Zr loading of 0.6 and 0.3 exhibited a high concentration of strong basic sites and, as a consequence, showed higher catalytic activity than the H-ZrCe1.2 catalyst. The obtained results revealed that the guarantee of low carbon deposition was due to the presence of Zr species in the lattice of periclase-like mixed oxides, which besides influencing basicity, also result in the formation of small Ni crystallites, inactive in direct methane decomposition and the Boudouard reaction [113].

Unlike Ce and Zr, La as a promoter can not only improve the anti-coke ability, but also enhance catalytic performance [104]. Side reactions such as methane decomposition were promoted at the same time; however, La can form oxycarbonate species and promote gasification of amorphous carbon deposits, resulting in lower carbon formation during the long-duration isothermal experiments performed at 550 °C. La-NiMgAl-LDO catalyst with 2 wt % La showed a 33% CH_4_ conversion at 550 °C, slightly higher than that of the un-promoted NiMgAl-LDO catalyst.

#### 3.1.3. The Types of Carbon Deposition

Carbon deposition is unavoidable during DRM and lead to catalyst deactivation. Different carbon species are formed according to the composition of catalysts and the reaction temperature. Thus, many researchers investigated the types of carbon formed on the catalysts’ surface. The main types of carbon were amorphous carbon, graphite, carbon nanotubes (CNTs) and carbon nanofibers (CNFs).

Amorphous carbon reflected no impediment to the DRM reaction [84], while encapsulated carbon was mainly responsible for nickel-containing catalysts’ deactivation, because the nickel crystallites were encapsulated by the carbon structures. Simultaneously, Daza et al. [94] also showed that different types of carbon formed at different temperatures. At 750 °C, carbon nanotubes (CNTs) and carbon nanofibers (CNFs) were formed, and the filamentous carbons were well-crystallized, yet displayed many structural defects, which increased the resistivity to fracture and prevented the encapsulation of active sites; while at 700 and 650 °C, carbon species were mainly graphite ribbons, coated carbon, nanoencapsulated graphite and Ni particles embedded inside the carbon were mainly responsible for the catalysts’ deactivation. Such types of carbon are deeply sensitive to reaction temperature. Düdder et al. [114] found that CNFs formed at 800 °C, and the formation was suppressed at 900 °C, whereas graphitic carbon formed at 900 °C; thus, CNFs are the most deactivating carbon species.

Plenty of works have been done on the application of LDH-derived catalysts to dry reforming of methane, and various LDH-derived catalysts have been developed. Dry reforming of methane was considered as the ideal type of reforming process, the main challenge being catalysts’ sintering, carbon deposition and applications to industrial applications. As “super-dry” reforming of methane has been developed, this process obtained higher purity H_2_, suppressed side reactions and coke deposition, saved energy compared with traditional dry reforming of methane and was promising for industrial application. Catalytic performance of dry reforming of CH_4_ for different catalysts in different works were summarized in Table 3.

### 3.2. Steam Reforming of Methane

Steam reforming of CH_4_ is the most common and generally the most economic way to produce H_2_. During the steam reforming of methane (MSR) process, there are two main reactions: steam reforming of methane (MSR) and the water gas shift reaction (WGS) [8,9]:

CH_4_ + H_2_O ↔ CO + 3H_2_ ΔH = 206 kJ/mol (9)

CO + H_2_O ↔ CO_2_ + H_2_ ΔH = −41kJ/mol (10)

**Figure 8:**
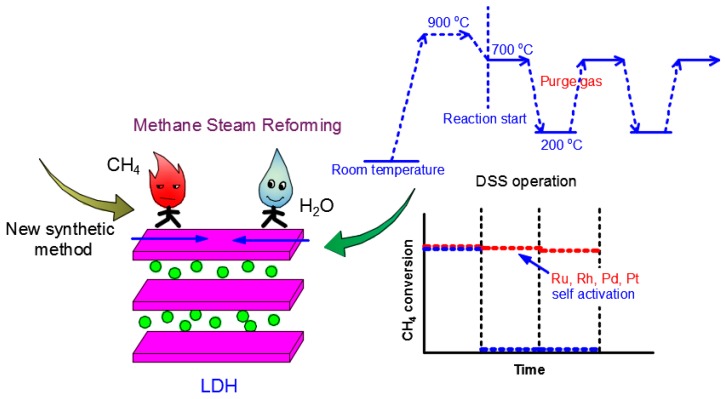
The application of LDHs in steam reforming of methane.

Because of the endothermicity of the reaction, conventional catalysts have the disadvantage of easy carbon deposition, though a high steam to carbon (S/C) ratio can be used to inhibit carbon formation, and the production costs is high. LDHs like catalysts show higher anti-coke and anti-sintering ability than conventional alumina-supported catalysts (Figure 8) [72,117].

Ni/Al LDH-derived catalyst was used as the MSR catalyst. Comas et al. [118] noticed that both reactants CH_4_ and H_2_O competed for the same active site of Ni during the SMR reaction, and CH_4_ conversion presented a maximum or decreased when the water feed concentration increased. When 20 mg of catalyst were used, the CH_4_ conversion reached a maximum of 46% at H_2_O/CH_4_ = 4. Nickel-supported LDHs displayed higher resistance to coke formation than the conventional Ni/γ-Al_2_O_3_ and Ni/CaO-Al_2_O_3_ catalysts, due to smaller Ni crystals showing a larger saturation concentration level of filamentous carbon than larger Ni crystals, which lead to the smaller driving force for carbon diffusion [117]. Ni-based LDH-derived catalyst composed by the co-precipitation route exhibited stronger metal-support interaction than that prepared by the incipient wetness method and gave smaller Ni crystals [119]. Catalyst prepared by co-precipitation exhibited high activity and excellent stability: for the 40 Ni/HT catalyst with 40 wt % Ni and Ni dispersion this was 10.8%, showing a 56% CH_4_ conversion and no obvious decrease after 25 h of reaction at 650 °C, much higher than that of commercial catalyst, which displayed 1.5% Ni dispersion was and 10% CH_4_ conversion. Dehghan-Niri et al. [120] reported that the spatially confined Ni nanoparticle inside a cage of porous ribbons had a relatively long distance between themselves, providing significant anti-sintering ability, which brought about a much lower deactivation rate than the commercial Ni catalyst.

Solid phase crystallization (spc) was also a valuable route to prepare highly efficient and eminent catalyst compared with the incipient wetness method [121]. The as-prepared spc-Ni_0.5_/Mg_2.5_-Al catalyst formed NiO-MgO solid solution during the thermal treatment, resulting in well-dispersed Ni metal particles on the catalyst. The spc-Ni/Mg-Al catalyst achieved a 60% CH_4_ conversion, and no decline in the activity was observed after 560 h.

In addition, electrodeposition was a new and an eminent method in LDH-derived catalyst preparation. Basile et al. [122] introduced and developed a Ni/Al-NO_3_ LDH-derived catalyst through electrodeposition in a single step on FeCr-alloy foams. Furthermore, the catalytic performance was greatly affected by the deposition time. The exHT-1.2-1000 (ex: after calcination) catalyst prepared under 1000 s and −1.2 V showed the best catalytic performance. However, the maximum CH_4_ conversion reached the equilibrium value of 67%, due to small and uniform particles of LDHs being deposited on the surface. An eggshell-type Ni/Mg-Al catalyst prepared by replacing a part of the Mg^2+^ by Ni^2+^ using the “memory effect” of LDHs’ structure showed an enhanced catalytic performance compared with impregnated catalyst [123]. The same catalyst formed “worm-like” structures and eventually constituted a dense Ni^2+^ layer and covered the surface of the particles, leading to enrichment of active Ni species. S-spc-Ni_0.51_/Mg_2.63_Al (eggshell-type Ni loaded catalysts) catalyst exhibited the best catalytic performance and achieved a 98.1% CH_4_ conversion at 800 °C, with a GHSV = 1.8 × 10^5^ mL h^−1^g^−1^. Takeguchi et al. [124] analyzed the coke formation of a nickel-based LDH-derived catalyst during steam reforming of methane. The coke deposition rate of the nickel-based LDH-derived catalyst was one third compared with the Ni/Al_2_O_3_ catalyst because Ni can be oxidized to a Ni-incorporated LDH structure by water-vapor treatment, and the deposited coke was easily removed by the reaction with oxygen in Ni-incorporated LDH [125].

Besides Ni, Ru, Cu and Co were also used as active components. The 1 wt % Ru supported by Co_6_Al_2_ oxide achieved an ~92% CH_4_ conversion at 600 °C, due to the reducible ruthenium and cobalt oxide species at the surface of the support with Ru adulteration [126]. When the reaction temperature reached to 700 °C, the catalyst displayed 100% CH_4_ conversion. Cu as an active component showed slightly lower catalytic performance than Ru, but also was an effective active ingredient. Moreover, Homsi et al. [8] studied the influence of copper content on the catalytic performance. The 5Cu/Co_6_Al_2_ catalyst with 5 wt % Cu showed the best catalytic performance and achieved a 96% methane conversion at 650 °C. With the increasing copper content, the catalyst activity decreased due to the formation of agglomerated and less reactive CuO species. The 5Cu/Co_6_Al_2_ catalyst can also achieve 100% methane conversion when the reaction temperature reached 700 °C. Lucr’edio et al. [127] used Co as the active component and studied the influence of the preparation method on the catalytic performance. Catalysts prepared by the traditional technique (traditional co-precipitation method; cobalt nitrate used as the Co ion source) and the anion-exchange method showed good activity, maintaining around 80% conversions during 6 h of reaction. While catalyst prepared by the co-precipitation method (cobalt complex chelate used as the Co ion source) showed an initial fall in conversion, it then remained around 40%, which was caused by cobalt active sites’ partial oxidation.

When catalyst is extensively used at the industry scale and operated at daily startup and shutdown (DSS) conditions, the catalyst bed must be purged by a sufficiently inert and an economically viable gas to prevent Ni metal from being oxidized, which could lead to deactivation [128]. Nonetheless, Ohi et al. [129] probed three kinds of purge gas and found that air as the purge gas led to quick deactivation of the oxidized surface metal Ni; spent gas was the most inert for the DSS operation and caused no significant deactivation; as the most convenient purge gas has a great relation to the (Mg + Ni)/Al ratio in Ni/Mg(Al)O catalysts, the most stable operation was achieved with a ratio of 6/1, while the (Mg + Ni)/Al ratio of 3/1 being the most prolific for the steady state operation validated the evident deactivation due to Mg(Al)O being hydrated by steam to form Mg(OH)_2_, resulting in the oxidation of Ni metal. Ni_0.5_/Mg_2.5_(Al)O catalyst achieved a 91% CH_4_ initial conversion, sharply decreasing to 45% under the first shut down, with deactivation after four cycles of the DSS operation [130]. However, when Ru was introduced to Ni_0.5_/Mg_2.5_(Al)O, the catalytic performance was effectively preserved under DSS operating conditions. Ru was introduced to the “memory effect” and formed Ru-Ni alloy, which had a strong interaction and effectively suppressed the deactivation. Besides, only 0.05 wt % of Ru loading was enough to suppress the deactivation effectively during the DSS-like operation. In follow-up work [131,132,133], the author researched the effect of other noble metals such as Rh, Pd and Pt in DSS operating conditions. In addition, Pd was not effective enough compared with Rh addition, since deactivation can be observed. Nevertheless, Rh and Pt with a loading of 0.05 wt % were effective at enhancing the stability of Ni_0.5_/Mg_2.5_(Al)O catalyst. The enhancement of stability under DSS operating conditions of Ru, Pt and Rh can be attributed to self-activation of the noble metal-Ni bimetal catalyst: the noble metal rather kept the reduced state during the steam purging and dissociated CH_4_ to form hydrogen atoms after the temperature reached 700 °C, then hydrogen atoms migrated to the oxidized Ni species by spillover and reduced them to the active Ni metals.

In conclusion, more research should be performed on the LDH-derived catalyst, for it holds promise as catalysts or supports of the SRM catalyst. Since SRM was the most common way to produce H_2_, more high-efficiency catalyst that show excellent catalytic performance and anti-coke ability should be explored. Moreover, reaction mechanism also can be researched to help design higher efficiency catalyst. Catalytic performance of steam reforming of CH4 for different catalysts in different works were summarized in Table 4.

### 3.3. Partial Oxidation of Methane

Catalytic partial oxidation of methane (POM), a mild exothermic process operated at short contact times, offered the greatest potential to synthesis of gas or hydrogen [10]. POM has become the focus of researches due to its obvious advantages, such as mild exothermicity, high energy efficiency and suitable CO/H_2_ ratio for methanol synthesis, and could be conducted in small reactors ideal for decentralized applications [11,12]. The equation is as follows:

CH_4_ + 1/2O_2_ → CO + 2H_2_ ΔH = −35.5 kJ/mol (11)

**Figure 9:**
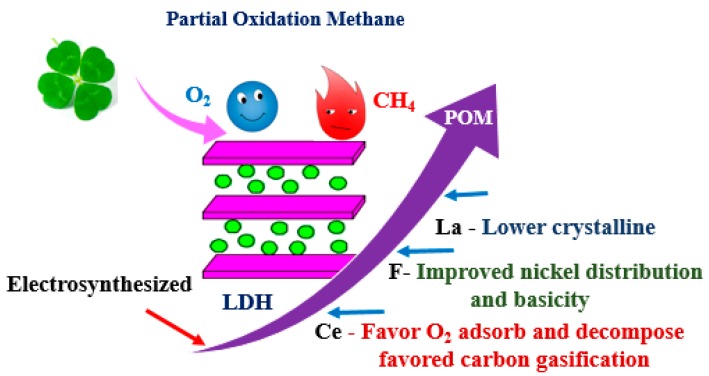
The application of LDHs in partial oxidation of methane. POM, partial oxidation of methane.

LDH-derived catalysts are also suitable catalysts for the POM reaction, as shown in Figure 9. Rh-based catalysts are very active in the POM, and Basile et al. [135,136,137,138] explored Rh containing LDH-derived Rh/Mg/Al catalysts, which showed better catalytic performance compared with supported Rh/A1_2_O_3_. Rh/Mg/Al catalysts with a metal ratio of 5.0/71.0/24.0 achieved 91% CH_4_ conversion at 750 °C. Rh-based LDH-derived catalysts also have been electro-synthesized on a FeCrAlY foam through the cathodic reduction of a solution containing metal salts and KNO_3_, the best catalytic performance being achieved by the catalyst obtained from the HT precursor prepared at −1.3 V for 1000 s. The coating of as-obtained catalyst RhexHT-1.3 pH has a high adhesion to the surface, exhibiting the best catalytic performance with a 90% CH_4_ conversion at 750 °C.

Even though Rh catalysts were very active in the POM of methane, the reduced availability and high cost of Rh could make it unsuitable for widespread commercial applications. Ru was less expensive than Rh and was active in the conversion of CH_4_. Ballarini et al. [139] unveiled the role of the composition and preparation method in the activity of LDH-derived Ru catalysts in the catalytic partial oxidation of methane. Both Ru dispersion and the interaction with the support decreased as the Ru loading increased and when silicates were present due to RuO_2_ segregation, and the 0.25 wt % Ru/Mg/Al-CO_3_ catalyst exhibited the best performance due to an enhanced metal-support interaction, carbon resistance and thermal stability. The 0.25 wt % Ru/Mg/Al-CO_3_ catalyst achieved 92% CH_4_ conversion and almost 100% CO selectivity at 750 °C with a volume ratio of CH_4_/O_2_/He = 2/1/20. Simultaneously, Velasco et al. [140] also found that with Ru addition, the catalyst showed excellent carbon resistance ability compared to the monometallic nickel catalysts. Harada et al. [141] used Ba_1.0_Co_0.7_Fe_0.2_Nb_3-δ_ (BCFN) dense ceramic supported by Mg-Al compound as an oxygen-permeable membrane for partial oxidation of methane; in 300 h, the oxygen permeation flux BCFN remained greater than 20 mL/(cm^2^ min). With the combination of the oxygen-permeable membrane, Ru 2 wt %/MgAlO_x_ catalyst afforded the best oxygen permeation performance and achieved initial methane conversion of 85%. This was the first report of such a high flux performance for a reaction in 300 h.

Catalysts with lower Ni content activated after a severe reduction treatment can show high stability during the reaction, while catalysts with high Ni content required mild reduction conditions and deactivated rapidly with time-on-stream due to carbon formation. The synergetic effect of Rh and Ni in Rh/Ni LDH-derived catalysts can increase the reducibility of Ni due to Rh being able to catalyze methane reacted with oxygen and increasing the surface temperature at the beginning of the bed [137]. 

La_2_O_3_ had a beneficial effect on the nickel dispersion, and the catalysts promoted with lanthanum toward CO_2_ reforming methane presented good conversion levels and lower carbon formation than unprompted catalysts. Thus, it was also a potential promoter of the POM reaction. Zhang et al. [10] found that the addition of La lowered the phase crystallization with the formation of small oxide particles. The as-prepared Ni/Mg/Al/La mixed oxides had both high activities and stabilities, the catalyst containing 6.5 mol % La showing the highest performance at 800 °C with a CH_4_ conversion of 99%, a CO selectivity of 93% and a H_2_ selectivity of 96%, which could be attributed to the presence of highly dispersed nickel and then the resistance to coke formation due to the promotion effect of lanthanum. Besides lanthanum, another rare-earth metal, cerium, also can increase the CO selectivity and decrease the carbon formation rate. With the cerium addition, oxygen was favorable to adsorbtion and decomposition with respect to these promoted catalysts, which favored the gasification of carbon species [142]. La- and Ce-promoted catalyst showed slightly lower catalytic performance, but achieved a higher CO selectivity than the un-promoted catalyst. The reason for higher selectivity was that La and Ce increased the surface basicity with consequent carbon reduction, which favored the dissociative adsorption of the oxygen in these catalysts [142].

Transition metals and rare-earth metals as promoters have been widely studied; non-metallic elements as the promoter have also displayed a positive effect. Zhang et al. [20] successfully introduced F into Ni-Mg-Al mixed oxide via the high dispersion of MgF_2_, which led to the formation of the periclase-type catalyst with a mesoporous structure. Fluorine-modified catalyst showed a low surface area and small Ni particle size, but high-moderate and strong basicity and exhibited excellent catalytic performance for POM without deactivation even after a 120-h run at 800 °C. The high catalytic performance resulted from F^−^ anions improving the homogeneous distribution of nickel and the basicity of the catalyst with high resistance to coking and sintering. Ni/Mg/AlO-F catalyst achieved almost 100% CH_4_ conversion at 800 °C. 

Cobalt has also been used as the active component in the POM reaction. Choudhary and Mamman found that at a molar ratio of CH_4_:O_2_ = 4:1, the CoO-MgO and NiO-MgO catalysts presented similar results at 700 °C [143]. Additionally, Lucre’dio et al. [142] utilized CoMgAl-Ht as the catalyst for the POM reaction, and the as-prepared catalyst displayed about 50% CH_4_ conversion at 750 °C, which reached equilibrium. From existing research, LDH-derived catalysts were propitious for POM. Even though POM required a lower amount of thermal energy compared with DRM and SRM, it required pure oxygen, which may lead to a danger with two combustible reagents. Catalytic performance of steam reforming of CH_4_ for different catalysts in different works were summarized in Table 5.

### 3.4. Autothermal Reforming

Autothermal reforming (ATR) is the combination of SMR and POM reactions, and the general reaction for ATR is described in Equation (12) [144]:

CH4 + ½xO_2_ + yCO_2_ + (1 − x − y)H_2_O ↔ (y + 1)CO + (3 − x − y)H_2_ (12)

It has low-energy requirements due to the opposite contribution of the exothermic methane oxidation and endothermic steam reforming; thus, it can avoid the necessity of a large external supply of heat and the cost of oxygen/nitrogen separation [13]. The combination of these two reactions can improve the reactor temperature, control and reduce the formation of hot spots and avoid catalyst deactivation by sintering or carbon deposition. Moreover, the H_2_/CO ratio of syngas produced by ATR has a wider range through manipulating the relative concentrations of H_2_O and O_2_ in the feed [14,15].

Ni- and/or Rh-containing LDHs have been used as catalysts for autothermal reforming of methane (in the presence or absence of ethane). NiRh alloy particles were formed in NiRh/MgAl, which was enriched in Ni [13]. NiRh/MgAl catalyst hardly catalyzed coke formation during CH_4_ autothermal reforming and exhibited excellent stability due to H_2_ spillover from Rh in the NiRh alloy against Ni oxidation. NiRh/MgAl catalyst displayed a 93% CH_4_ conversion at 500 °C [13]. Luneau et al. [144] tested the long-term stability of a series of catalysts in a six parallel-flow reactor and found that the 5–0.05 wt % Ni-Rh/MgAl_2_O_4_ catalyst was robust for the autothermal reforming of model biogas, because Rh can effectively promote nickel reduction and prevent bulk oxidation. Likewise, Souza et al. [145] researched Ni-Mg-Al LDH-derived catalysts with varied Ni content for CH_4_ autothermal reforming. All catalysts exhibited only MgO-periclase phase X-ray diffraction peaks, suggesting that both nickel and aluminum were well dispersed in the MgO matrix. The performance of LDH-derived catalysts was very similar, with a CH_4_ conversion of about 85%, without any apparent deactivation during the stability test at 800 °C. All catalysts achieved a maximum CH_4_ conversion of 94% at 900 °C.

Besides the co-precipitation method, the solid-phase crystallization method (spc) was an effective method to prepare high efficiency catalysts and has been used to prepare catalysts for the DRM and SRM reaction. The spc-Ni_0.5_/Mg_2.5_Al catalyst with a ratio of Mg/Al of 1/3 showed excellent autothermal reforming performance [146]. Meanwhile, Ni dispersion was further enhanced during the spc preparation process. The spc-Ni_0.5_/Mg_2.5_Al catalyst attained almost a 97.5% CH_4_ conversion at 800 °C and showed no deactivation during the 50-h stability test. In order to obtain high purity H_2_, LDH-derived catalyst also used CO_2_ as the sorbent in sorption-enhanced autothermal reforming of methane. Combined with a traditional Ni/MgO catalyst, CH_4_ conversion was enhanced to 99.5% with a H_2_ purity of 99.5%, higher than that without LDH-derived CO_2_ sorbent: 85% and 96%, respectively [147,148].

Autothermal reforming (ATR) was energy saving, and the H_2_/CO ratio ranges between one and two [7]. Thus, autothermal reforming was also a good choice to produce syngas. Since LDH-derived catalysts displayed excellent catalytic performance and LDH-derived CO_2_ sorbent applied to sorption-enhanced autothermal reforming can produce higher purity H_2_, thus more attention should be paid to this research, and new efficient catalysts should be further explored. Catalytic performance of autothermal reforming for different catalysts in different works were summarized in Table 6.

## 4. Conclusions

As conventional 2D materials, LDHs showed small crystal sizes, high surface area, compositional flexibility, memory effect and basic properties, and the as-obtained catalysts displayed large surface area, high thermal stability and a high extent of dispersion of metallic particles after reduction, so being considered as suitable supports or catalysts for CO/CO_2_ methanation and the methane reforming reaction.

(1) Methanation

The NiAl-LDH catalyst with a high Ni loading showed good catalytic performance, but poor anti-sintering ability. The introduction of dopants can effectively decrease the Ni content, improve the reducibility and enhance the interaction between nickel and LDH-based supports, thus further improving the catalytic performance and anti-sintering ability. However, the research of LDH-derived catalysts in the methanation reaction was inadequate. As promising catalysts for the methanation reaction, much further work will be necessary, especially for the single-layer LDHs. The atomic monolayers benefit from the high-speed transfer of the carrier in the material, show similar wrinkles as for graphene and favor the dispersion of the active component as monodispersed nanoparticles, which have great potential in single-atom catalysts.

(2) Methane reforming

Similar to the methanation reaction, the NiAl-LDH catalyst has been also used for the methane reforming reaction, and plenty of work has been performed to restrain the coke deposition. Dopants (Rh, La, Ce, C, etc.) can improve the anti-coke ability of catalysts by improving the active dispersion, enhancing the interaction between the active component and the support and increasing the surface basic sites. Different synthesis methods have also been studied. Besides, the morphology also evidently influences the anti-coke ability, and the monolithic and egg-shell catalysts have shown better anti-coke ability in the dry-reforming reaction. They are also promising catalysts in other kinds of methane reforming.

In addition, the “memory effect” synthesis scheme is also a favorable method for the preparation of highly dispersed LDH-derived catalysts for both methanation and methane reforming reactions. So far, although some dopants have been introduced, the diversification of LDH-derived catalysts is not sufficient, and multicomponent LDH-derived catalysts should be explored.

## Figures and Tables

**Table 1:** Catalytic performance of CO methanation for different catalysts in different works.

Catalyst	Ni (wt %)	Temperature (°C)	Pressure (MPa)	GHSV (mL g^−1^ h^−1^)	CO Conversion (%)	CH_4_ Selectivity (%)	Ref.
NiMg8	11	550	0.1	15,000 h^−1^	99.8	73.6	[48]
Ru/NiAl-C	30	150	0.1	2400 h^−1^	100	--	[42]
NiAl-LDO	50 mol %	400	0.1	300,000	100	90	[35]
Ni/VMT-LDO	10	400	1.5	20,000	87.9	90	[34]
30Ni10FeAX	30	230	1	8160	99.4	79.6	[46]
25Fe75Ni	5	275	--	50,000 h^−1^	100	99.1	[42]

**Table 2:** Catalytic performance of CO_2_ methanation for different catalysts in different works.

Catalyst	Ni (wt %)	T (°C)	Pressure (MPa)	GHSV (mL g^−1^ h^−1^)	CO_2_ Conversion (%)	CH_4_ Selectivity (%)	Ref.
Ni/Mg-Al	59	330	0.1	66,000	74	--	[56]
Ni-Al 12	76	300	0.1	20,000 h^−1^	86	86 (yield)	[21]
NiFeAl-(NH4)_2_CO_3_	30	220	1	9600	58.5	99.5	[55]
Ni_21_La_0.4_(IE)	21	300	--	12,000 h^−1^	80	99.4	[58]
HTNi	20	150	0.1	20,000 h^−1^	80	80 (yield)	[60]
Ni-Ce-LDH-P	~4	270	--	60,000	75	100	[61]

**Table 3:** Catalytic performance of dry reforming of CH_4_ for different catalysts in different works.

Catalyst	Ni (wt %)	T (°C)	GHSV (mL g^−1^ h^−1^)	CO_2_/CH_4_ Ratios	CH_4_ Conversion (%)	Ref.
Ru/Mg_3_(Al)O	2	800	60,000	1	84	[71]
NiMgAl-700	10	800	8000	1	95	[79]
Mg_5_NiAl_2_O_9_	20	850	7200 h^−1^	1.25	95.8	[72]
NiMgAl-LDO/γ-Al_2_O_3_	9.6	700	24,000	1	80.7	[83]
NiO-MgO-Al_2_O_3_	13.47	800	5000	1.0/1.1	91	[82]
Ru/Co_x_Mg_y_Al_2_	--	750	--	1	97	[105]
spc-Ni/Mg-Al	25.1	800	54,000	1	94.5	[81]
Ni/CeO_2_-ZrO_2_/MgAl_2_O_4_	15	850	5000	0.4	81	[115]
La-NiMgAlO	2.8	750	48,000	1	90	[91]
CeO_2_-Ni/MgAl_2_O_4_	12	850	5000	0.4	86.2	[116]
Ni/Mg/Al/Ce	48.03	700	48,000	1	80	[92]
Ce-Ni/Mg-Al	50 mol %	700	48,000	92.3	89.4	[94]
Ce-Ni/Mg/Al	50 mol %	800	30,000	100	95	[97]
HS-550	10 (NiO)	800	12,000	1	85%	[101]
HT-NiAl	63.5	550	20,000 h^−1^	2	48	[108]
HT-100Ni	58.66	550	20,000 h^−1^	1	55	[110]
H-18NiCe	17.9	550	20,000 h^−1^	1	41	[111]
HTNi-CeZr	19.3	550	20,000 h^−1^	1	25	[112]
NiLaMgAl	15	550	20,000 h^−1^	1	33	[103]
HT-NiMgA	5	750	--	1	87.5	[98]
IR-NiMgAl-LDH@m-SiO_2_	5.84	800	--	1	90	[99]
Co_2_Mg_4_Al_2_HT500	25 mol %	800	--	1	96	[104]
Ni-Mg-Al-nano-spheroid	15	800	--	1	95	[100]
12% Co/Mg_3_Al	12	800	60,000	1	90	[103]
R-C-350-1.8-800	10	800	48,000	1	80	[33]
1Co-2Ni-LDH	5	800	30,000	4/6	98.3	[106]

**Table 4:** Catalytic performance of steam reforming of CH_4_ for different catalysts in different works.

Catalyst	Ni (wt %)	T (°C)	Pressure (MPa)	GHSV (mL h^−1^g^−1^)	S/C Ratios	CH_4_ Conversion (%)	Ref.
40 Ni/HT	40	650	0.1	--	3	56	[119]
spc-Ni0.5/Mg2.5-Al	~9.8	740	0.1	2890	1.6	60	[134]
exHT-1.2-1000	1.2	900	2	--	1.7	67	[122]
s-spc Ni_0.51_/Mg_2.63_	8.2	800	--	180,000	2	98.1	[123]
Ru/Co_6_Al_2_	1 (Ru)	700	0.1	--	3	100	[126]
5Cu/Co_6_Al_2_	5 (Cu)	700	0.1	15,000	3	100	[9]
Ae-MgAl-CoY	12.5 mol % (Co)	750	--	49 h^−1^	2	80	[127]

**Table 5:** Catalytic performance of partial oxidation of CH_4_ for different catalysts in different works.

Catalyst	Ni (wt %)	T (°C)	Pressure (MPa)	GHSV (h^−1^)	CH_4_/O_2_ Ratios	CH_4_ Conversion (%)	Ref.
RhexHT-1.3pH	0.2 (Rh)	750	0.1	28,000	2	90	[140]
Ni/Mg/Al/La	21	800	--	--	2	99	[11]
Ru/Mg/AleCO_3_	0.25 (Ru)	750	--	--	2	92	[141]
CoMgAl-Ht	5 mol % (Co)	750	0.1	--	2	50	[142]
Ni/Mg/AlO-F	36 mol %	800	0.1	--	2	100	[26]

**Table 6:** Catalytic performance of autothermal reforming for different catalysts in different works.

Catalyst	Ni (wt %)	T (°C)	Pressure (MPa)	GHSV (mL g^−1^ h^−1^)	CH_4_/O_2_/H_2_O Ratios	CH_4_ Conversion (%)	Ref.
NiRh/MgAl	25	500	--	1,700,000	2/1/2	93	[13]
10NiHT	10	900	0.1	160 h^−1^	4/1/2	94	[145]
spc-Ni_0.5_/Mg_2.5_Al	16.3	800	--	150,000	2/1/2	~97.5	[146]
